# Archaeal Communities in Deep Terrestrial Subsurface Underneath the Deccan Traps, India

**DOI:** 10.3389/fmicb.2019.01362

**Published:** 2019-07-16

**Authors:** Avishek Dutta, Pinaki Sar, Jayeeta Sarkar, Srimanti Dutta Gupta, Abhishek Gupta, Himadri Bose, Abhijit Mukherjee, Sukanta Roy

**Affiliations:** ^1^Environmental Microbiology and Genomics Laboratory, Department of Biotechnology, Indian Institute of Technology Kharagpur, Kharagpur, India; ^2^School of Bioscience, Indian Institute of Technology Kharagpur, Kharagpur, India; ^3^School of Environmental Science and Engineering, Indian Institute of Technology Kharagpur, Kharagpur, India; ^4^Department of Geology and Geophysics, Indian Institute of Technology Kharagpur, Kharagpur, India; ^5^Ministry of Earth Sciences, Borehole Geophysics Research Laboratory, Karad, India; ^6^CSIR-National Geophysical Research Institute, Hyderabad, India

**Keywords:** deep biosphere, archaeal diversity, Deccan traps, Illumina sequencing, metabolism

## Abstract

Archaeal community structure and potential functions within the deep, aphotic, oligotrophic, hot, igneous provinces of ∼65 Myr old basalt and its Archean granitic basement was explored through archaeal 16S rRNA gene amplicon sequencing from extracted environmental DNA of rocks. Rock core samples from three distinct horizons, basaltic (BS), transition (weathered granites) (TZ) and granitic (GR) showed limited organic carbon (4–48 mg/kg) and varied concentrations (<1.0–5000 mg/kg) of sulfate, nitrate, nitrite, iron and metal oxides. Quantitative PCR estimated the presence of nearly 10^3^–10^4^ archaeal cells per gram of rock. Archaeal communities within BS and GR horizons were distinct. The absence of any common OTU across the samples indicated restricted dispersal of archaeal cells. Younger, relatively organic carbon- and Fe_2_O_3_-rich BS rocks harbor *Euryarchaeota*, along with varied proportions of *Thaumarchaeota* and *Crenarchaeota*. Extreme acid loving, thermotolerant sulfur respiring *Thermoplasmataceae*, heterotrophic, ferrous-/H-sulfide oxidizing *Ferroplasmaceae* and *Halobacteriaceae* were more abundant and closely interrelated within BS rocks. Samples from the GR horizon represent a unique composition with higher proportions of *Thaumarchaeota* and uneven distribution of *Euryarchaeota* and *Bathyarchaeota* affiliated to *Methanomicrobia*, SAGMCG-1, FHMa11 terrestrial group, AK59 and unclassified taxa. Acetoclastic methanogenic *Methanomicrobia*, autotrophic SAGMCG-1 and MCG of *Thaumarcheaota* could be identified as the signature groups within the organic carbon lean GR horizon. Sulfur-oxidizing *Sulfolobaceae* was relatively more abundant in sulfate-rich amygdaloidal basalt and migmatitic gneiss samples. Methane-oxidizing ANME-3 populations were found to be ubiquitous, but their abundance varied greatly between the analyzed samples. Changes in diversity pattern among the BS and GR horizons highlighted the significance of local rock geochemistry, particularly the availability of organic carbon, Fe_2_O_3_ and other nutrients as well as physical constraints (temperature and pressure) in a niche-specific colonization of extremophilic archaeal communities. The study provided the first deep sequencing-based illustration of an intricate association between diverse extremophilic groups (acidophile-halophile-methanogenic), capable of sulfur/iron/methane metabolism and thus shed new light on their potential role in biogeochemical cycles and energy flow in deep biosphere hosted by hot, oligotrophic igneous crust.

## Introduction

The Earth’s deep continental biosphere is hot, oligotrophic, and limited by several physical and chemical constraints for life; yet it harbors up to 19% of the Earth’s biomass (Whitman et al., 1998; McMahon and Parnell, 2014; Kieft, 2016). Exploration of microbial life in such extreme habitat represents one of the most intriguing sectors with enormous opportunities for reconnoitering the evolution and adaptation of life on our planet and beyond (Colwell and D’Hondt, 2013). In spite of considerable research attention, the deep biosphere is still counted among the “least known frontiers of Earth” and our effort to generate a global census on deep microbial life remains limited by habitat accessibility (Breuker et al., 2011; Purkamo et al., 2018).

Despite the harsh conditions in terms of lack of readily utilizable energy and nutrient sources, extreme pressure and temperature, limited space and fluid mobility, microbial life has been observed across diverse deep crystalline biosphere settings (Fredrickson and Balkwill, 2006; Edwards et al., 2012; Miettinen et al., 2015; Kieft, 2016). It is interesting to note that the inhabitant microorganisms of such extreme habitats not only evolve striking adaptive strategies to sustain their living, but may also require those conditions for their survival (Rampelotto, 2013; Kieft, 2016). These organisms are often referred as extremophiles and include members of all three domains of life, i.e., *Bacteria*, *Archaea*, and *Eukarya*, of which archaea represents a small but important proportion. Nevertheless, they are proficient in adapting to different extreme conditions including survival under chronic energy stress and holding different “extremophily records” (Swan and Valentine, 2001; Rampelotto, 2013) and are the important contributors to subterranean biogeochemical cycles (Biddle et al., 2006; Silver et al., 2012; Offre et al., 2013; Magnabosco et al., 2016). Archaea are among the most hyperthermophilic, acidophilic, alkaliphilic, and halophilic microorganisms known. Previous studies have provided valuable information on the distribution and composition of archaea within deep igneous terrestrial subsurfaces and their role in carbon, nitrogen and sulfur geocycling (Offre et al., 2013; Ino et al., 2018). Archaea mediated subsurface H_2_ cycling, methanogenesis, anaerobic methane oxidation, iron and sulfur redox transformation, and nitrate reduction are crucial components of subsurface lithoautotrophic microbial ecosystem (SLiME) and/or mixotrophic ecosystems (Stevens and McKinley, 2000; Nealson et al., 2005; Gregory et al., 2019; Smith et al., 2019). Because of their extremophilic nature, significance in different biogeochemical cycles and syntrophic relations with bacterial members, archaea are considered as suitable residents of deep subterranean environments.

However, compared to several investigations highlighting bacterial diversity, focused studies on archaea in deep terrestrial subsurface are very limited (Takai et al., 2001; O’Connell et al., 2003; Waldron et al., 2007; Kieft, 2016; Lazar et al., 2017; Purkamo et al., 2018). One of the first studies which focused solely on archaea in deep terrestrial subsurface was carried out in South African gold mines (Takai et al., 2001). The study revealed “great” phylogenetic diversity and highlighted novel lineages of the phyla *Crenarchaeota* (SAGMCG-1 and SAGMCG-2) and *Euryarchaeota* (SAGMEG-1 and SAGMEG-2). More recently, SAGMCG was reclassified into the phylum *Thaumarchaeota* (Brochier-Armanet et al., 2008). The presence of niche-specific archaeal communities was observed within subsurface organic rich sedimentary rocks of Antrim Shale, United States (Waldron et al., 2007; Wuchter et al., 2013). The prevalence of lithoautotrophic archaea within carbonate-rock/siliciclastic-rock aquifer systems and their restricted dispersal from the surface to the subsurface environment was reported by Lazar et al. (2017). The widespread occurrence of methanogenic and anaerobic methane-oxidizing archaea (ANME); members of more versatile *Thermoplasmatales* are reported from deep granitic environment of the Witwatersrand deep subsurface, Chelungpu fault, Gifu prefecture, and from Precambrian bedrock fluids in Fennoscandian Shield (Takai et al., 2001; Moser et al., 2005; Gihring et al., 2006; Bomberg et al., 2015; Wu et al., 2015; Ino et al., 2016; Purkamo et al., 2018 and references within). Methanogenesis and methane cycling processes are a major contribution of archaea in deep crystalline rock biosphere, which are considered to be closely interconnected with redox transformation of electron acceptors (nitrate, sulfate, metal oxides, and humics) or electron donors (hydrogen, ammonia, nitrite, and sulfide) (Kietäväinen and Purkamo, 2015; Ino et al., 2018; Smith et al., 2019) and thus play significant roles in overall community function. A few studies have profiled the archaeal diversity in the deep terrestrial biosphere, but their distribution and metabolic significance in biogeochemical cycles within deep subsurface igneous provinces remains poorly explored. The hard rock terrestrial deep biosphere in basalt, metabasalt and granitic horizons have so far mainly been explored by analyzing the groundwater; studies of rock samples recovered through scientific deep drilling are rare (Breuker et al., 2011 and references within; Hubalek et al., 2016; Kieft, 2016; Ino et al., 2018).

Here, we aim to explore the archaeal communities hosted by ∼65 Ma old basalts and underlying Archean basement granitoids comprising the deep terrestrial subsurface igneous province of the Deccan traps province using the cores recovered through scientific deep drilling (Roy et al., 2013; Gupta et al., 2015). The Deccan traps are a massive continental flood basalt province (total area > 0.5 million km^2^) resting on ∼2.5 Ga Archean crystalline basement (Sen, 2001; Schoene et al., 2015; Bhaskar Rao et al., 2017). The Koyna–Warna region of Deccan traps is known for recurrent reservoir triggered seismicity following the impoundment of the Koyna dam in 1962 (Gupta, 1992, 2017). Exploratory deep drilling at multiple sites in the Koyna seismogenic zone allowed us to access the deep subsurface rock cores covering the entire thickness (varying between 500 and 1250 m) of Deccan basalt (designated as BS), a few 100 m of the underlying granitic basement (GR) down to 1400 m depth and an intermediate region of weathered granites (referred as transition zone or TZ) between BS and GR horizons. The goal of this study is to shed light on the potential archaeal communities across the different depths of deep basaltic-granitic subsurface. This work provides a detailed information on archaeal life in deep igneous terrestrial subsurface where extreme temperature and pressure regimes (on average 25°C increase per 1000 m in basalt and 15°C increase per 1000 m in granite; 26.7 MPa increase in lithostatic pressure per 1000 m) and geochemical conditions have shaped the environment in a unique way (Roy and Rao, 2000; Dutta et al., 2018).

## Materials and Methods

### Sample Collection

Subsurface core samples were collected from different depths of two exploratory drill holes at Ukhalu (N 17° 07.552′, E 073° 52.148′) and Phansavale (N17° 09.017′, E073° 40.058′) of Koyna–Warna region of Deccan Traps, Maharashtra during July 2014 and May 2015. Eleven samples were obtained to cover the three major horizons: Deccan basalt (BS) (∼65 Ma), the underlying granitic basement rock (GR) (∼2500 Ma), and the short section between the two formations representing the weathered surface of granitic basement affected by the first lava flows (referred here as a transition zone, TZ) (see Supplementary Table S1). Samples were checked for the presence of sodium fluorescein (500 mg m^–3^) used during drilling (Nyyssönen et al., 2014). Only interior pieces of rock were selected for microbiological study to avoid potential drilling mud contamination as recommended elsewhere (Lever et al., 2013). Samples were collected following aseptic techniques and stored in sterile containers at 4°C for shipment. In the laboratory, the samples were stored at −20°C to limit the chance of microbial contamination until further analysis.

### Rock Processing

Rock cores were washed thoroughly under sterile condition with autoclaved, DNA free water (Thermo Fisher Scientific^®^) and sub-coring of the rock was done to get rock samples devoid of any possible contamination that might have occurred during drilling. At first, the uneven ends of the cores were removed using granite cutter fitted with sterilized cutting blade. Subsequently, with either a mechanical drilling instrument fitted with sterilized drill bit or with sterilized chisel and hammer, rock powder was obtained from the interior core. Sub-coring was done in different layers and sodium fluorescein was checked in the sub-cored rock powder. Rock powders were dissolved in water and aqueous fraction was used for detection of fluorescein using spectrophotometric analysis (Mota et al., 1991). The rock powders free from sodium fluorescein were stored in sterilized DNA free containers at −20°C for further geochemical and microbial analysis.

### Geochemical Analysis

Rock powders were sieved through 2 mm mesh. Acid digestion of these rock powders was conducted using HNO_3_ and HF (3:1) in a microwave digester (Milestone SK12) for efficient extraction of the elements from rock powder to aqueous media followed by the measurement of major elements using Inductively Coupled Plasma Mass Spectrometry (iCAP Q, Thermo Fisher Scientific). Rock powders were ultrasonicated for major anions (Cl^–^, NO_2_^–^, SO_4_^2–^, NO_3_^–^, and PO_4_^3–^) using Dionex ICS2100 (Thermo Fisher Scientific). Total organic carbon (TOC), total inorganic carbon (TIC) and total carbon (TC) were measured using an OI analytical TOC analyzer. Before analyzing TOC, rock powders were fumigated with 1N HCl for 48 h under a fume hood. Quantification of the elemental oxides was done using a PANalytical Epsilon3 XRF instrument. Alkalinity of the rocks was measured using the USEPA method 310.2. The pH of the rock samples (in the presence of water) was measured by incubating 1 g of rock powder in 0.1 M CaCl_2_ at 1:10 ratio (w/v), and highly sensitive probes (Orion) fitted with an Orion multi-meter (Thermo Electron Corporation, Beverly, MA) were used (Islam et al., 2014). Four out of the eleven rock core samples used in this study (two each from borehole; U9, U8, PV4, and PV2) were used previously for a geomicrobiological investigation (Dutta et al., 2018). Geochemical properties of these four samples were included in this study for a better comparison (along with seven other samples).

### Extraction of Environmental DNA

Community DNA was extracted from 11 samples using the MoBio power soil DNA isolation Kit (MoBio) following the manufacturer’s protocol. Total DNA from reagent control was also extracted using the same procedure as mentioned earlier, and were used subsequently to check any possible contamination. The quality (purity) and quantity of the extracted environmental DNA was first determined using a NanoDrop 2000 spectrophotometer, followed by precise fluorometric quantitation using Qubit (Thermo Fisher Scientific).

### Quantitative Polymerase Chain Reaction (qPCR)

Quantification of the archaeal populations in the rock samples were performed by estimating the copy number of archaeal specific 16S rRNA gene. The abundance of methanogenic populations was estimated from *mcr*A copy numbers using quantitative PCR (qPCR). Details of qPCR primers used are provided in Supplementary Table S2. Two μl of extracted environmental DNA were added to the PCR mastermix with a total volume of 10 μl. All the reactions were set in triplicates. Quant Studio 5 was used to perform qPCR with Power SYBR green PCR mastermix (Invitrogen), with primer concentration of 5 pM and the following amplification conditions: 95°C for 10 min, 40 cycles of 95°C for 15 s, 55°C for 30 s and 72°C for 30 s. Melting curve analysis was run after each assay to check PCR specificity. Genes encoding archaeal 16S rRNA and *mcr*A were PCR amplified from the extracted environmental DNAs, cloned in TA cloning vector and 10-fold standard dilution series of plasmid DNAs with 10^2^–10^10^ copy numbers of 16S rRNA and *mcr*A were used to quantify both genes in the environmental samples.

### 16S rRNA Gene Amplification and Sequencing

Following quality checks, the extracted environmental DNAs were sent to the Marine Biological Laboratory, Woods Hole, United States for Illumina MiSeq sequencing using the procedures outlined on the VAMP website^1^. Archaeal V4-V5 hypervariable region of 16S rRNA gene was sequenced on Illumina MiSeq platform. The primers used for amplification of archaeal V4-V5 region are 517F and 958R (Supplementary Table S2). The sequence reads were submitted to short read archive under SRA accession: SRP126374.

### Bioinformatic Analysis

Paired-end reads obtained from Illumina MiSeq sequencing were merged into single end reads using FLASH (Magoč and Salzberg, 2011) with a minimum and maximum overlap of 10 and 100, respectively and a mismatch density of 0.25. This step was followed by quality filtering using the quantitative insights into microbial ecology (QIIME 1.9.1) (Caporaso et al., 2010). Sequences having lengths outside bounds of 250 and 500, mean quality scores below a minimum of 25 and maximum homopolymer runs exceeding a limit of 6 were filtered out. *De novo*-based clustering of reads to form OTU was performed using UCLUST under QIIME workflow. Sequences having greater than 97% similarity were assigned to the same OTU. Representative reads from each OTU were assigned taxonomy using UCLUST trained SILVA 128 database (Quast et al., 2012). OTUs which were present in the reagent controls were removed from OTU pool of the samples. Alpha diversity parameters were calculated using alpha_diversity.py under QIIME workflow.

### Statistical Analysis

Selected geochemical parameters across 11 samples were used to perform principal component analysis (PCA) using PAST software (Hammer et al., 2001). Geochemical parameters were normalized on the basis of feature scaling before PCA was plotted. Archaeal families from across the samples were selected for Spearman correlation analysis and correlation heat-map was constructed using METAGENassist (Arndt et al., 2012). The abundant archaeal classes were used to construct PCA biplot using PAST software. Non-metric multidimentional scaling (NMDS) analysis of Bray–Curtis metric based on all the archaeal families was performed using PAST software, and 21 different physico-chemical parameters were declared as environmental variables. Similarity percentage (SIMPER) analysis was also done using PAST software for 11 samples to identify the microbial families responsible for the grouping of different rock types. OTU overlap among the samples from similar rock type and OTU pool of different rock types was elucidated using InteractiVenn (Heberle et al., 2015).

### Network Analysis

Co-occurrence network analysis was performed to ascertain major connections among archaeal populations and different geochemical parameters of the samples. Correlation among different archaeal genera with geochemical parameters was calculated using the otu.association command in mothur (Schloss et al., 2009). Pairwise Spearman correlations with correlation significance value <0.05 was used for the construction of co-occurrence networks using Cytoscape 3.4.0 (Shannon et al., 2003). Two separate networks were constructed for positive and negative correlations.

### Prediction of Functionality of Archaeal Communities

Metagenomic inventories of the archaeal communities were predicted using PICRUSt (Phylogenetic Investigation of Communities by Reconstruction of Unobserved States) (Langille et al., 2013). Closed referenced based OTU picking using Greengene 13.5 as a reference database was performed in QIIME, and PICRUSt was used to allocate the functional attributes by comparing the identified 16S rRNA gene sequence with the nearest match of the known genome sequence. A weighted nearest sequenced taxon index (NSTI) was calculated to evaluate the accuracy of prediction of the metagenomes. A PCA biplot was constructed on the basis of relative abundance of different metabolisms to understand the relatedness among different archaeal communities on the basis of their community functions.

## Results

### Geochemical Characteristics of the Samples

**FIGURE 1:**
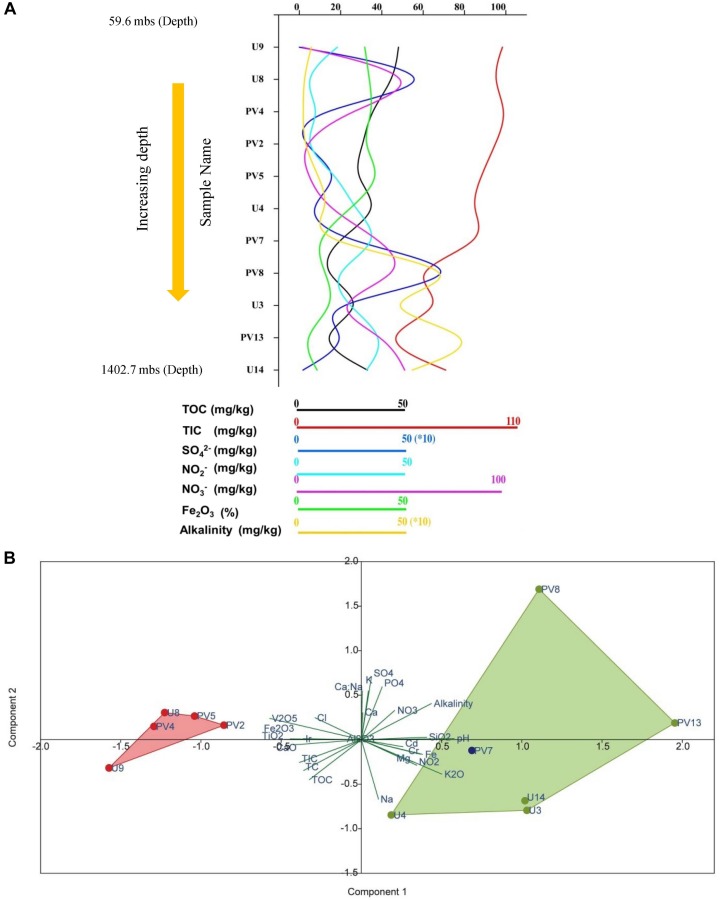
Distribution of different geochemical parameters and archaeal population across different samples of subterranean Deccan traps. **(A)** Variations of different geochemical parameters across samples. **(B)** Principal component analysis (PCA) of geochemical parameters of subsurface rock cores of Koyna–Warna region; Green dots represent samples from the granitic horizon, red circles represent samples from the basaltic horizon and blue dot represents sample from the transition zone. mbs – meters below surface.

Rock core samples obtained from varying depths across the basaltic layers of Deccan traps and underlying granitic basement were analyzed for major geochemical properties. Geochemical parameters pertinent to microbial activities were plotted (Figure 1A and Supplementary Table S3). The samples showed a characteristic trend in their geochemical properties with increasing depth. Based on these parameters, the distinct geochemical nature of granitic and basaltic horizons was evident, whereas samples from the transition zone showed a mixed nature. Concentrations of major anions like NO_3_^–^, and SO_4_^2–^; metals like Mg, Fe, Na, Cr, Cd, and oxides of Si and K increased with depth. Samples from shallower levels were distinctive based on slightly acidic pH, higher TOC, TIC, TC, CaO, TiO_2_, and Fe_2_O_3_. Although certain unexpected variations in geochemical parameters across different horizons were observed, PCA showed that the samples from two different (BS and GR-TZ) zones were geochemically distinctive with respect to the measured geochemical parameters (Figure 1B). Concentrations of TOC, TIC, and TC decreased with increasing depth and were generally lower in GR horizon compared to BS horizon samples. NO_3_^–^ and NO_2_^–^ were overall on the lower side when compared to SO_4_^2–^ whereas PO_4_^3–^ was below detection limit for all the samples except sample PV8.

### Archaeal Abundance in Deccan Subsurface

Archaeal 16S rRNA gene and *mcr*A gene copy numbers in the analyzed rock samples were quantified using real time PCR. 16S rRNA- and *mcr*A-gene copy numbers could be successfully obtained for four samples (three BS and one GR samples), while for the remainder of the samples, it was not successful even after repeated attempts (Table 1). The 16S rRNA gene copies varied between 2.14 × 10^3^ and 4.75 × 10^4^ per gram of rock (with an average copy number of 1.6 × 10^4^/gram of rock). Assuming an average occurrence of 1.7 copies of the 16S rRNA gene in archaeal genomes (Stoddard et al., 2015), we estimated an archaeal cell abundance ranging from 1.25 × 10^3^ to 2.78 × 10^4^ per gram of rock. Gene fragment of *mcr*A was observed in the same four samples and its copy number varied from 4.1 × 10^1^ to 1.6 × 10^3^ (with average copy number of 5.18 × 10^2^). Percentage of *mcr*A copy number with respect to archaeal cell abundance varied between 3 and 8% and was observed to be higher in GR sample (8%) over the BS samples (average 4%; Standard deviation 1%).

**TABLE 1:** Sequencing information and qPCR data.

**Sample name**	**U9**	**U8**	**PV4**	**PV2**	**PV5**	**U4**	**PV7**	**PV8**	**U3**	**PV13**	**U14**
Rock type	BS	BS	BS	BS	BS	GR	TZ	GR	GR	GR	GR
Total no. of raw reads	1630	2484	3829	5052	7001	4159	3867	963	1348	2176	4893
Quality filtered reads	1044	1245	3237	4909	6079	3861	2331	693	1315	2025	4078
Total no. of final reads	1031	1245	2901	4176	5718	1523	1528	469	300	436	1243
Observed OTUs	153	152	520	531	549	427	348	140	191	226	441
Number of unique OTUs	111	99	381	436	440	360	248	85	154	160	359
% of unique OTUs	73	65	73	82	80	84	71	61	81	71	81
Singles	108	116	357	394	437	329	267	88	144	172	323
Doubles	17	9	59	52	37	34	42	15	23	20	34
Shannon	3.82	3.75	5.98	4.81	4.35	6.12	4.98	5.80	7.18	6.97	7.22
Simpson	0.79	0.81	0.94	0.86	0.88	0.93	0.87	0.96	0.99	0.98	0.97
goods_coverage	0.90	0.91	0.88	0.91	0.92	0.78	0.83	0.81	0.52	0.61	0.74
Chao1	474	819	1579	1992	3056	1969	1174	379	620	926	1927
Archaeal cell count	1.25E+03	2.78E+04	ND	5.37E+03	ND	ND	ND	2.93E+03	ND	ND	ND
*mcrA* copy number	4.11E+01	1.57E+03	ND	2.12E+02	ND	ND	ND	2.47E+02	ND	ND	ND

*ND – not detected. All the cell counts and copy numbers are measured per gram of rock. BS – Basalt; TZ – Transition; GR – Granite.*

### Sequencing Data

**FIGURE 2:**
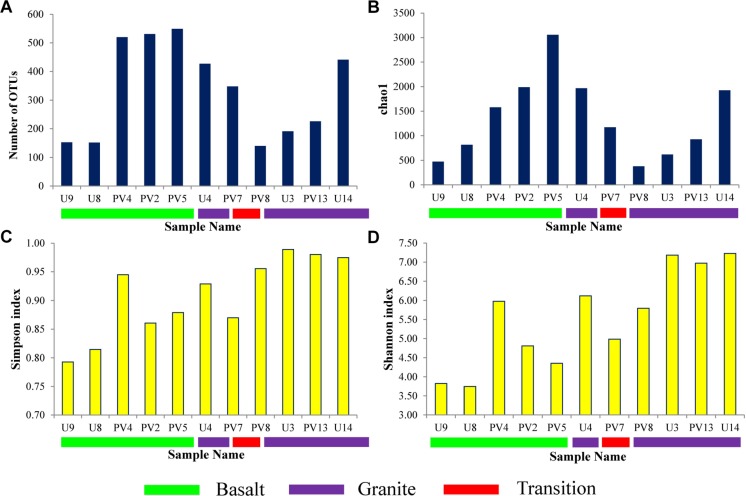
Alpha diversity parameters of different samples. **(A)** Observed OTUs, **(B)** Chao1 estimator, **(C)** Simpson Index, and **(D)** Shannon Index.

Sequence information and diversity indices are summarized in Table 1. Illumina MiSeq based sequencing of the hypervariable V4-V5 region of archaeal 16S rRNA gene produced 32509 numbers of total merged reads for 11 samples. After filtering and removing potential erroneous reads, 26739 reads were used for OTU picking. A maximum of 6079 and a minimum of 693 filtered reads were obtained for PV5 and U3, respectively. OTUs containing reads from reagent (DNA extraction) control were removed from the OTU pool. A total of 20570 reads grouped into 3148 OTUs were finally obtained with a maximum of 549 (PV5) and a minimum of 140 (PV8) OTUs. Out of these total OTUs, only 6.5% were unassigned to any taxa. Noticeably, 2833 unique OTUs (∼90% of the total OTUs) were found across the samples and these OTUs represent 31% of the total reads. Interestingly, the number of observed OTUs increased with depth in basalts followed by a reduced copy number in the transition zone. Similarly, the number of OTUs increased with depth in granites except for sample U4 (Figure 2A). A comparable trend was observed in the Chao1 index used to estimate the minimal number of OTUs present. The Chao1 index suggests the presence of a maximum of 3056 OTUs and a minimum of 379 OTUs in PV5 and PV8, respectively (Figure 2B). Simpson’s (measures species dominance) and Shannon’s (evaluates species abundance and evenness) indices, ranged from 0.79 to 0.99 and 3.75 to 7.22 and both increased with depth (Figures 2C,D). Overall, the average Shannon diversity index was much higher in granites than in basalts indicating higher species richness in granites.

### Archaeal Community Analysis

**FIGURE 3:**
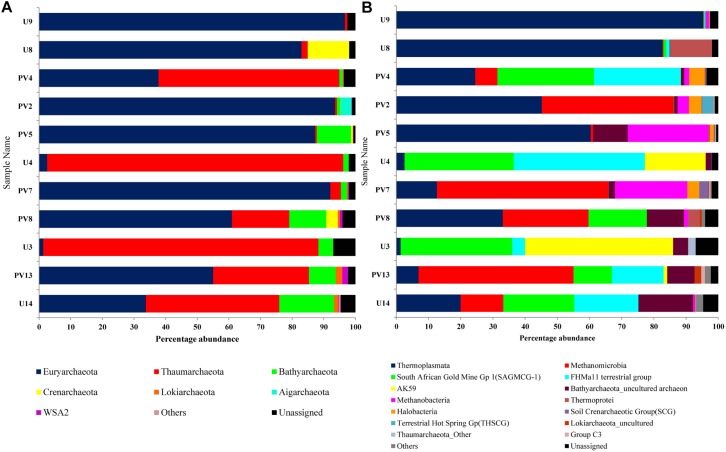
Distribution of archaeal population across different samples of subterranean Deccan traps. Archaeal community composition of Deccan subsurface at **(A)** phylum level and **(B)** class level.

16S rRNA gene sequences assigned to archaea covered 9 phyla, 12 classes, 7 orders, 20 families, and 40 genera. *Euryarchaeota* was the most abundant phylum (average abundance of 59%) followed by *Thaumarchaeota* (30%) and *Bathyarchaeota* (5%) (Figure 3A). *Crenarchaeota*, *Lokiarchaeota* and WSA2 were sporadically observed across different samples. *Aigarchaeota* and pMC2A209 were only found in sample PV2 (BS) whereas Marine Hydrothermal Vent Group (MHVG) was observed in sample U14 (GR). A comparison between BS and GR samples revealed that the former had considerably higher proportion of *Euryarchaeota* (average 80%), but lower proportion of *Thaumarchaeota* (average 12%) and *Crenarchaeota* (average 2%). In GR, although the members of *Euryarchaeota* were detected at moderately high abundance (31%), *Thaumarchaeota* outnumbered and represented the most abundant taxa (54%). *Bathyarchaeota* present as a minor group in BS (2.6%), constituted 8.7% in GR.

**FIGURE 4:**
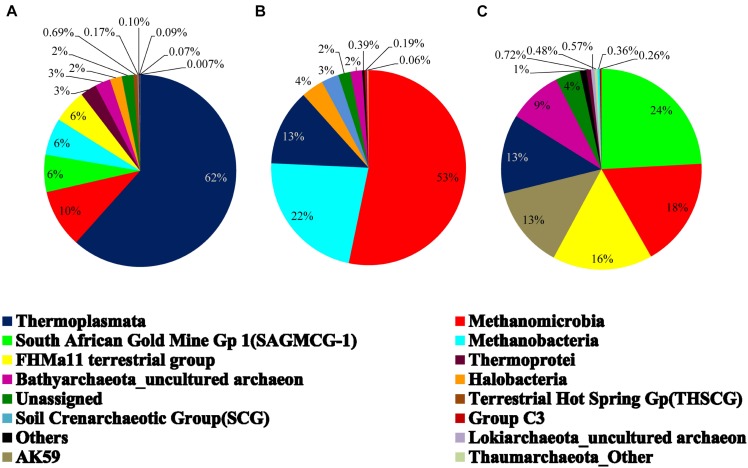
Average archaeal community composition of **(A)** basalt, **(B)** transition, and **(C)** granite at class level. Since only one sample from the transition zone was selected, percentage abundance of the archaeal community is shown.

On the basis of cumulative abundance, *Thermoplasmata*, *Methanomicrobia*, South African Gold Mine Gp 1(SAGMCG-1), FHMa11 terrestrial group and AK59 were identified as the five major archaeal classes (Figure 3B). *Thermoplasmata* (*Euryarchaeota*) was much more abundant in BS- (62%) than GR-cores (13%). In contrast, average abundance of three *Thaumarchaeota* members namely the SAGMCG-1, FHMa11 terrestrial group and AK59; as well as *Methanomicrobia* (*Euryarchaeota*) were more abundant in the GR cores (24, 16, 13, and 18%, respectively) than in BS samples (6, 6, 0, and 10%, respectively) (Figure 4). Other archaeal classes detected in the cores recovered from the Deccan subsurface were *Methanobacteria*, *Thermoprotei*, *Halobacteria*, Soil Crenarchaeotic Group (SCG), and Terrestrial Hot Spring Gp (THSCG). Among these, *Methanobacteria* (*Euryarchaeota*) and *Thermoprotei* (*Crenarchaeota*) were relatively more abundant in BS (average abundance of 6 and 3%, respectively) compared to GR (0.4 and 0.7%). Other archaeal classes detected in this study were present only in the BS samples. Significant presence of *Methanomicrobia* (53% abundance), *Methanobacteria* (22%) and *Thermoplasmata* (13%) was observed in TZ core. *Halobacteria* (*Euryarchaeota*) and SCG (*Thaumarchaeota*) furthermore comprised more than 1% of the total reads in the TZ core. Methanogenic archaea were present across the samples and horizons with slightly higher average abundance in GR (18%, BS: 16%). Four out of seven known methanogenic archaeal orders were present in the order of highest to lowest abundance in GR: *Methanosarcinales*, *Methanomicrobiales*, *Methanobacteriales*, and *Methanocellales*. This order of abundance was different in the BS horizon where *Methanobacteriales* was the most abundant followed by *Methanomicrobiales, Methanosarcinales*, and *Methanocellale*. In general, samples from the Phansavale site harbored more methanogenic taxa (particularly for *Methanomicrobia* and *Methanobacteria*).

Taxonomic affiliation at the lowest level (genus) was possible only for 62% of total classified reads (44% of the OTUs). *Thermoplasma* followed by *Methanosaeta*, *Ferroplasma*, *Methanobacterium*, and *Methanolacinia* represented the five most abundant genera across the samples. Interestingly all these major genera belonged to the phylum *Euryarchaeota*. Other archaeal genera detected in greater than 1% cumulative abundance were *Methanolobus*, ANME-3, *Methanolinea*, *Methanocalculus*, *Candidatus Nitrososphaera*, *Natronorubrum*, *Methanosarcina*, *Methanocorpusculum*, *Methanospirillum*, *Methanogenium*, *Methanoregula*, and *Halovivax*. The distribution of the major genera showed a distinct preference for either of the horizons, although a few, like ANME-3 and Terrestrial Miscellaneous Gp (TMEG) were present in a greater number of samples with varying abundance (0–4.5 and 0–1.6%, respectively). Higher average abundance of *Thermoplasma* (35%), *Ferroplasma* (21%), *Methanobacterium* (6%) and *Methanolacinia* (4%) was observed in BS. In GR horizon, *Methanosaeta* was more dominant (14%, compared to 3% in BS) along with several unclassified taxa affiliated to *Bathyarchaeota*, AK59, FHMa11 terrestrial group and SAGMAG-1 (average abundance: 9–24%).

### Endemic Archaeal Population in Different Rock Types

**FIGURE 5:**
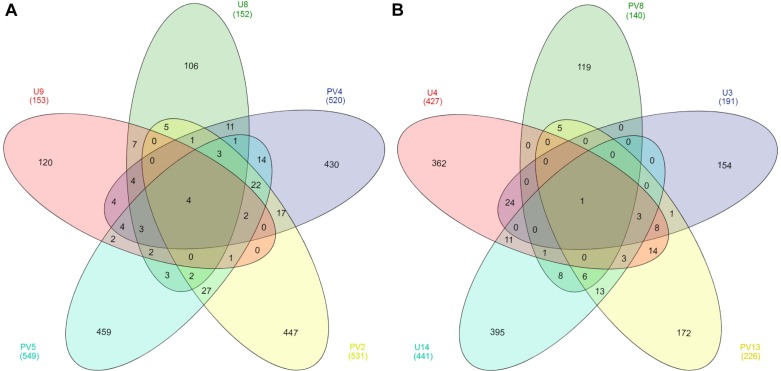
Venn diagram of OTUs present in **(A)** basaltic and **(B)** granitic zones. OTUs were based on 97% sequence similarity. The numbers inside the diagram are the number of OTUs.

Out of a total of 3148 OTUs only four core OTUs (OTUs present in all samples) were observed across all the BS samples whereas only one core OTU was observed for the GR horizon (Figure 5). Core OTUs of BS and GR represent 12.59 and 0.13% of total archaeal reads, respectively. Of the four core OTUs of BS samples, three were affiliated to *Ferroplasma* and one to the genus *Thermoplasma*. The single core OTU detected in GR horizon was affiliated to SAGMCG-1. No shared OTUs were observed across all the samples from BS and GR horizons. From a total pool of 2900 OTUs observed across all the BS and GR samples, only 3.5% of the OTUs were present in both the horizons whereas 55% and 41% of OTUs remained unique to BS and GR horizons (Supplementary Figure S1).

### Correlation Studies and Deciphering the Niche-Specific Colonization of Archaeal Taxa

**FIGURE 6:**
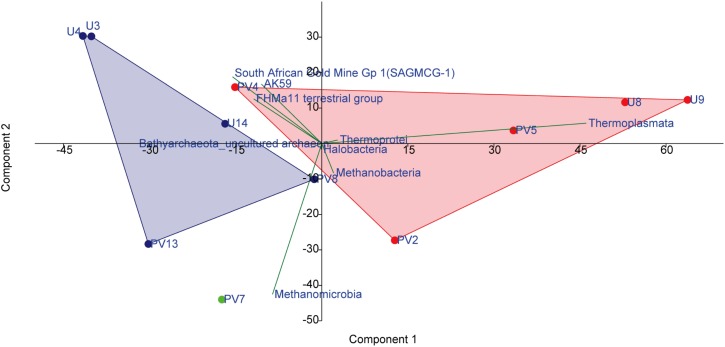
PCA analysis on the basis of the abundance of archaeal classes present among different samples. Red symbols represent basaltic samples; green symbol represents transition zone sample; blue symbol represents granitic samples.

A set of statistical tools were used to investigate the nature of correlation and the niche-specific colonization of archaeal populations within the BS and GR horizons. A PCA biplot was drawn on the basis of abundance of different archaeal classes which showed distinct assemblages of archaea across BS and GR horizons. As evident in Figure 6, regardless of the borehole sites, subsurface archaeal communities partitioned into (i) BS zone guild and (ii) GR zone guilds. Close association among *Thermoplasmata*, *Thermoprotei*, *Methanobacteria* and *Halobacteria* members in BS cores, and SAGMCG-1, FHMa11 terrestrial group, AK59 and an unclassified class of *Bathyarchaeota* (which represented the dominant archaeal assemblage within the GR horizon) in GR cores was observed. Sample from transition zone (PV7) did not group with any of the other groups and remained distinctly placed. One of the BS samples, PV4, clustered with the rest of the BS samples, but showed relatively higher similarity with the GR communities.

**FIGURE 7:**
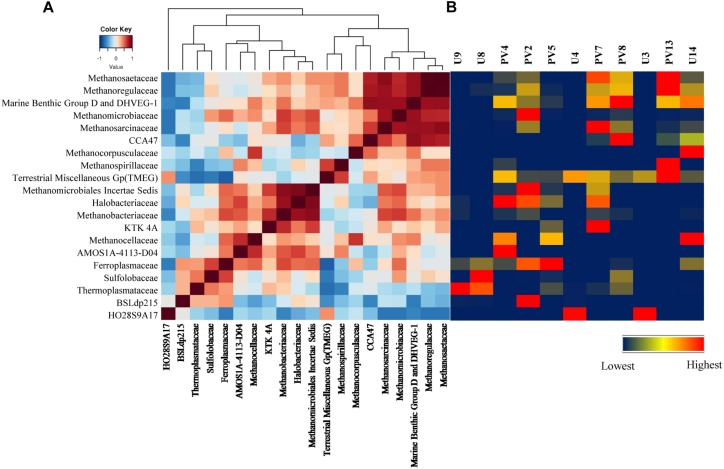
Correlation among different microbial classes. **(A)** Heatmap of Spearman correlation across archaeal families. **(B)** Heatmap of relative percentage abundance of corresponding archaeal classes across all the samples.

Correlation among the archaeal populations was identified through the Spearman correlation method. Based on correlation values, two distinct groups/clusters each consisting of 9 and 10 archaeal families were observed (Figure 7A). Cluster 1 was exclusively represented by *Euryarchaeota* members and could be divided in to two distinct sub-clusters. One of the sub-clusters consisted of *Methanosaetaceae*, *Methanoregulaceae*, Marine Benthic Group D and DHVEG-1, *Methanomicrobiaceae* and *Methanosarcinaceae* whereas the other sub-cluster comprised of CCA47, *Methanocorpusculaceae*, *Methanospirillaceae* and TMEG. Most of the families in this cluster are known methanogens (both hydrogenotrophic and acetoclastic types). Interestingly, these families were more abundant in GR rock cores with relatively low contributions in BS samples (Figure 7B). Cluster 2 consisted of 10 families, of which nine of the members were affiliated to *Euryarchaeaota*. This cluster comprised of two sub-clusters of which one consisted of *Methanomicrobiales Incertae Sedis*, *Halobacteriaceae*, *Methanobacteriaceae*, KTK 4A, *Methanocellaceae*, AMOS1A-4113-D04, and *Ferroplasmaceae*. The second sub-cluster represented a strong relation among *Sulfolobaceae*, *Thermoplasmataceae*, and BSLdp215. This group consists of archaeal families known to be thermophilic/thermotolerant, methanogens, and participate in the sulfur cycle. Their presence was mainly observed in BS cores (Figure 7B).

**FIGURE 8:**
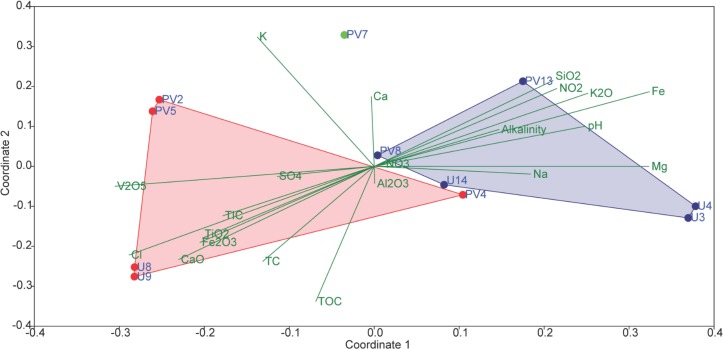
Non-metric multidimensional scaling (NMDS) analyses of microbial communities and its association with environmental factors of subsurface rock cores of Koyna–Warna region (Bray–Curtis, Stress = 0.12). Red symbols represent basaltic samples; green symbol represents transition zone samples; blue symbols represent granitic samples.

In order to assess the collective role of environmental variables on archaeal populations inhabiting the BS and GR horizons NMDS (using archaeal families and set of environmental parameters) based analysis was performed; which separated the samples into two distinct abundance-weighted categories (Figure 8). Vectors in NMDS displayed the major environmental parameters which might govern the unique archaeal assemblages in BS and GR horizons. SIMPER analysis was used to determine the archaeal taxa responsible for the differences observed between the BS and GR archaeal microbiomes (Supplementary Table S4). The topmost “discriminating” taxa were *Thermoplasmataceae*, *Ferroplamaceae*, and *Methanosaetaceae*. Average taxonomic distribution patterns demonstrated that BS microbiomes contained a higher relative abundance of *Thermoplasmataceae*, *Ferroplasmaceae*, *Methanobacteriaceae*, *Methanomicrobiaceae*, *Sulfolobaceaea*, AMOS1A-4113-D04, and *Halobacteriaceae* compared to GR. On the other hand, GR microbiome displayed greater relative abundance of *Methanosaetaceae*, Marine Benthic Group D and DHVEG-1 and unassigned families of SAGMCG-1, AK59, FHMa11 terrestrial group and *Bathyarchaeota*.

### Archaeal Co-occurrence and Effect of Geochemical Parameters

**FIGURE 9:**
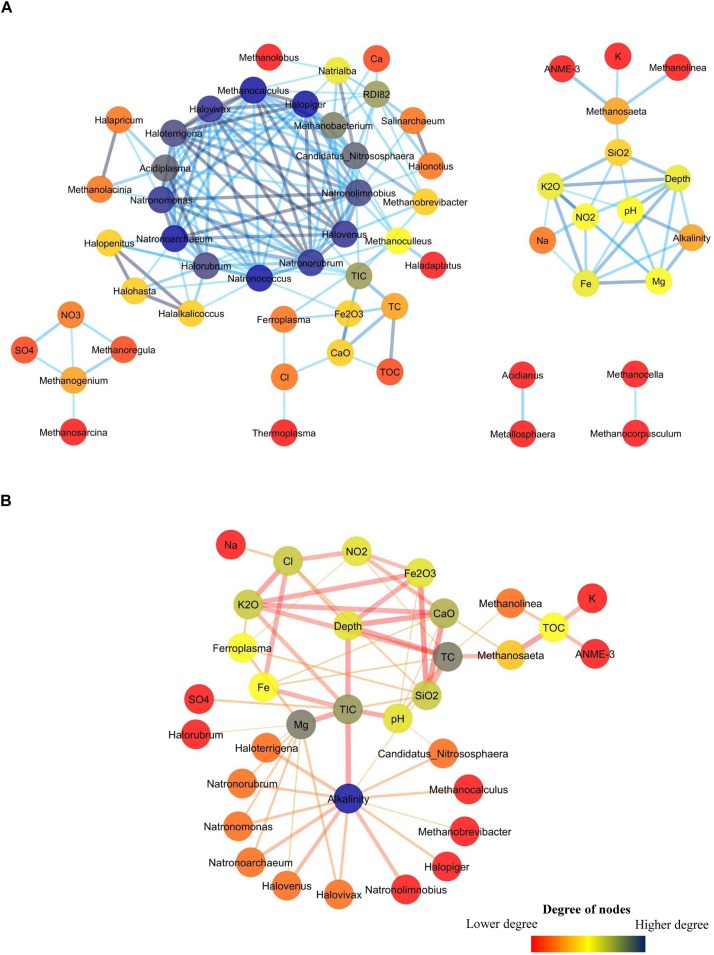
Co-occurrence network among archaeal genera and geochemical parameters in subsurface rock cores of Koyna–Warna region. The color of the nodes represent the number of degrees it has. **(A)** Network showing positive correlation. **(B)** Network showing negative correlation.

A network analysis was done to better understand the co-occurrence among different environmental parameters and archaeal taxa in Deccan subsurface. Networks for positively and negatively co-related archaeal genera with different environmental parameters were separately constructed. The positively correlated network consisted of 58 nodes and 178 edges (Figure 9A) whereas the negatively correlated network had fewer nodes (33) and edges (62) (Figure 9B). The positively correlated network consisted of five sub-networks. The main sub-network consists of a hotspot (hotspots are region where large numbers of connections are observed among nodes) constituted by 14 members. This hotspot was mainly dominated by halotolerant archaea viz. *Halovivax*, *Haloterrigena*, *Natrorubrum*, and others. This cluster also includes members of acidophilic *Acidiplasma*, methanogenic *Methanobacterium* and *Methanocalculus* and ammonia-oxidizing *Candidatus Nitrososphaera*. Though most of these archaeal members are present in very low abundances, it is interesting to note that these organisms were mainly present in higher depth basalts (PV4, PV2, and PV5) and transition zone of the Phansavale borehole. The peripheral nodes of the hotspot consisted of archaeal genera observed mainly in the BS cores. Of these peripheral nodes and edges, one of the significant connections was between acidophilic iron-oxidizing *Ferroplasma* and Fe_2_O_3_. Sub-network consisting of *Methanosaeta*, *Methanolinea*, and ANME-3 also included other geological parameters, which had an increasing trend with depth. Connections between *Methanolinea*, methanotrophic ANME-3 and other geochemical parameters was established by *Methanosaeta*, which harbors versatile capability of acetoclastic methanogenesis and is also known for its methylotrophic nature. Sub-networks comprising of *Methanoregula*, *Methanogenium*, and *Methanosarcina* were correlated with elevated concentrations of SO_4_^2–^ and NO_3_^–^. We also observed two micro-networks consisting of one connection each. One of the micro-networks consisted of sulfur-oxidizing *Metallosphaera* and *Acidianus* which were mainly found in amygdaloidal basalt (U8), which displayed elevated SO_4_^2–^ concentration while compared to other basalts. The second micro-network consisted of two hydrogenotrophic methanogens *Methanocella* and *Methanocorpusculum* whose co-presence was observed in the deepest recovered granitic core (U14).

The network showing negative correlation consisted of 33 nodes (Figure 9B). Alkalinity was the node having highest degree (13) and was connected to the groups which were mainly present in the hotspot of the positively correlated network. *Ferrolpasma* was negatively correlated with the geochemical parameters (Mg, SiO_2_, K_2_O, and NO_2_^–^) which were present in higher abundances in most of the granites. Interestingly, *Methanosaeta*, ANME-3 and *Methanolinea* were found to be negatively correlated with TOC.

### Predicted Metabolic Properties

**FIGURE 10:**
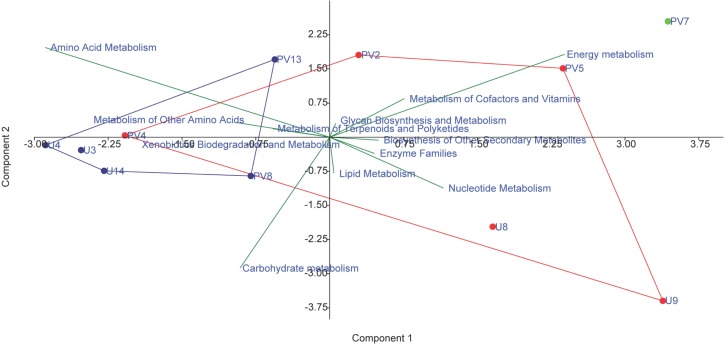
PCA biplot on the basis of the most abundant metabolism related to archaeal genes in the predicted metagenomes; red symbol depicts basaltic samples; blue symbol depicts granitic samples and green symbol depicts transition zone sample.

The metabolic properties of the archaeal communities hosted by the subsurface rocks from different horizons were predicted from 16S rRNA gene sequences using PICRUSt (Table 2). The NSTI was calculated for all the samples to evaluate the accuracy of prediction of the metabolic properties (Supplementary Table S5). The NSTI values varied between 0.05 (predicted microbiome for PV2, BS core) and 0.32 (predicted microbiome for U4, GR core). The most abundant metabolism displayed by the majority of the archaeal groups were amino acid metabolism (19–25% of all metabolism genes), carbohydrate metabolism (20–24%) and energy metabolism (13–18%). Genes allocated for amino acid metabolism and carbohydrate metabolism were elevated in GR samples (average 25 and 23%, respectively) compared to BS samples (average 22% for both) (Table 2). The PCA biplot was constructed on the basis of the relative abundance of different metabolisms to understand the grouping of the archaeal community function across different rock samples on the basis of predicted community function (Figure 10). Except for sample PV4 (a BS core recovered from a depth of 106.4 m below surface), all the basalts and granites formed distinct clusters. Predicted functions of the archaeal community hosted by sample PV4 were similar to the predicted metabolisms of the GR which was evident from the PCA. PV7 (TZ) did not cluster with BS or GS group showing distinct community metabolism. Similar grouping was observed in previous PCA and NMDS analysis which was carried out on the basis of archaeal classes and families, respectively. A similar trend obtained from different statistical analysis (on the basis of taxonomy and predicted community functions) (Figures 6, 8, 10) implies that taxonomy of different archaeal groups in particular microbiomes reflects the archaeal community function.

**TABLE 2:** The most abundant metabolism related to archaeal genes in the predicted metagenomes.

**Metabolism categories**	**U9**	**U8**	**PV4**	**PV2**	**PV5**	**U4**	**PV7**	**PV8**	**U3**	**PV13**	**U14**
Amino acid metabolism	19	21	25	23	22	25	22	23	25	25	24
Carbohydrate metabolism	24	23	23	20	21	24	21	23	24	22	23
Energy metabolism	15	14	14	14	17	14	18	14	14	16	13
Nucleotide metabolism	12	11	9	9	10	9	10	10	10	10	10
Metabolism of cofactors and vitamins	8	9	8	10	9	8	9	8	8	9	9
Xenobiotics biodegradation and metabolism	5	6	5	6	4	5	4	5	5	4	4
Lipid metabolism	4	4	4	4	3	3	3	4	3	3	4
Enzyme families	4	3	3	3	3	3	3	3	3	3	3
Metabolism of other amino acids	1	2	3	2	2	3	2	2	3	2	2
Biosynthesis of other secondary metabolites	2	2	2	2	2	1	2	2	1	2	1
Glycan biosynthesis and metabolism	1	1	1	2	2	1	2	1	1	1	2
Metabolism of terpenoids and polyketides	3	4	4	5	4	4	3	4	4	4	4

*All the values are in percentage.*

## Discussion

Deep subsurface of the Deccan traps province may be considered as an extreme environment for microbial life with several physical as well as chemical stressors. On average, temperature increases with depth at a rate of ∼25°C per km in the Deccan basalt formation and ∼15°C per km in the granitic bedrock (Roy and Rao, 1999, 2000). Typically, temperatures at a depth of 1 km are in the range 40–45°C and at 1.5 km, 55–60°C in the study region. Lithostatic pressure increases at a rate of ∼26.7 MPa per km. Nevertheless, the archaeal abundance in this extreme habitat is estimated to be in the range of 10^3^–10^4^ cells per gram of rock, well within the range (10^2^–10^5^ archaeal 16S rRNA gene copies per gram) reported earlier from several deep terrestrial subsurface ecosystems (Breuker et al., 2011; Bomberg et al., 2015; Purkamo et al., 2016). The broad geochemical spectrum of the rock cores portrays critical details of the abiotic components of this unique ecosystem and provides the perspective to understand its microbial ecology more meaningfully.

The biological, geochemical and hydrological processes at work within the deep terrestrial habitats in the Deccan traps and underlying basement rocks have likely influenced the archaeal community structure. Together with their gneissic/migmatitic nature, these rocks exhibit structural deformation and associated fractures, which could be connected to the seismicity of this region (Misra et al., 2017). Based on their geochemical properties, the three classes of rock samples could be distinctly separated; particularly the basaltic and granitic provinces. Although the overall Deccan subsurface was low in organic carbon content, relatively higher levels detected in the BS horizon could be linked to intrusion of surface organic matter, or remnants of the ancient organic matter derived from diverse past life forms present within the different layers of lava flows. The deeper GR horizon is more depleted in organic carbon, and this is in line with the extremely low porosity, lack of fluid mobility and perhaps low biological activities that might produce any “in house” organic matter (Goswami et al., 2017). Granitic rocks are formed deep within the continental crust by the cooling of intruding magma, which are often devoid of organic carbon. Unlike the scarcity of organic carbon, minerals present in deep igneous rocks are an important source of electron acceptors (iron, sulfate, nitrate/nitrite) available to the microorganisms present (Sen, 2014). Availability of sulfate in the deeper horizons (>1000 m) is widely reported through geochemical analysis of deep granitic aquifers around the globe (Ino et al., 2018 and references therein) and this corroborates well with the present study. Sulfate dependent oxidation of methane and other reduced substrates (e.g., hydrogen, ammonia, nitrite) have been identified as a potent metabolic driver in oligotrophic deep continental subsurface (Nealson et al., 2005). Scarcity of organic carbon but relative abundance of iron (as Fe_2_O_3_), oxidized nitrogen compounds and sulfate in the deep subsurface indicate the broad chemical landscape of this extreme igneous province that could potentially support a versatile microbial catabolic repertoire impacting the deep subsurface element cycling and biomass production.

A marked difference in species diversity and OTU distribution as observed with respect to the sample’s depth may be linked primarily to the local rock geochemistry and the *in situ* temperature and pressure conditions of the horizons. The dominance of fewer archaeal species in the shallower depth BS than in GR possibly signifies the role of specialist organisms capable of thriving in relatively nutrient wealthy niches. On the other hand, a diverse but more even community structure as detected within the GR highlights the existence of more generalist archaeal groups which may work in synergy for the optimal use of limited available nutrients. A previous study has indicated that the diversity and resilience of archaeal communities in low nutrient deep biospheres could be promoted by mutualistic interactions among community members or emergent properties resulting from their slow reproduction rates (Nyyssönen et al., 2014). Spearman correlation (inter-taxa), PCA and abundance weighted NMDS illustrate the community assemblages and the role of subsurface geochemistry (including Fe_2_O_3_, organic carbon, nitrate and sulfate) in shaping such assemblages. The significance of local geochemical and geophysical factors on microbial community structure and function in various crystalline deep biosphere has previously been reported (Brazelton et al., 2013; Magnabosco et al., 2014; Ino et al., 2018).

Distribution patterns of archaeal taxa, correlation and network analyses clearly indicate the mutual relationship among the taxa highlighting the niche-specific colonization of specific populations within the BS and GR horizons. In general, a basalt centric archaeal community in younger basaltic rocks with relatively higher concentrations of organic carbon, iron oxide and sulfate could be delineated. This was distinct from the community assemblage within the organic carbon lean, deeper, Archean granitic rocks. At lower taxonomic level these basalt and granitic centric community composition illustrated the possible road maps of subsurface biogeochemistry, carbon flux and microbial (archaeal) ecology.

The basalt centric archaeal community of acidophilic, halophilic and methanogenic populations each with their varying abundances portrays the major archaeal guild and provides a possible clue to energy and carbon flows within the younger and relatively organic carbon rich basaltic rocks. Thermoacidophilic *Thermoplasma*, *Ferroplasma*, and *Sulfolobaceae* members abundant here are well known resident of Fe, S rich deep subsurface extreme environments with or without tectonic, volcanic and thermal activities (Golyshina, 2011, 2014; Reysenbach and Brileya, 2014; Heberle et al., 2015; Ijiri et al., 2018; Arce-Rodríguez et al., 2019). *Thermoplasma* and *Ferroplasma* both lack cell wall, and are heterotrophic, facultative aerobic, extreme (often obligate) acidophiles (pH 0.0–1.0) with optimum growth temperature at around 60°C. *Thermoplasma* gains energy anaerobically from sulfur respiration and produce H_2_S, while *Ferroplasma* has been recognized with its “ubiquitous capacity” for ferrous iron oxidation and a strict dependence on low concentration of yeast extract (Macalady et al., 2004; Golyshina, 2011, 2014; Reysenbach and Brileya, 2014). The simultaneous presence of both *Thermoplasma* and iron oxidizing *Ferroplasma* has been reported recently from acidic, iron-rich deep mine environment of Pyhäsalmi Mine, Finland (Bomberg et al., 2019). Significant connections between *Ferroplasma* and Fe_2_O_3_ in the major network hotspot corroborate their niche-specific localization. Together with Fe-oxidizing and S-respiring populations, the presence of *Sulfolobaceae* is also noticed in basalts. *Sulfolobaceae* members are known to adapt simultaneously with high temperature fluctuations (>60°C to <100°C) and low pH (<4.0), and they are equipped with diverse chemolithotrophic, heterotrophic, and mixotrophic metabolism (utilizing broad range of carbon sources including amino acids, simple to complex substrates) (Albers and Siebers, 2014). Based on the known physiological and metabolic properties of these archaeal taxa, we could infer that in iron-sulfur rich BS rocks of elevated temperature, thermoacidophiles allow extraction of energy through their chemolithotrophic and/or dissimilatory metabolism (coupled to heterotrophy) and thus generate biomass and metabolic intermediates for other members of this ecosystem. Together with these thermophilic taxa, presence of sparsely populated alkaliphilic *Halobacteriaceae* members detected exclusively in the BS samples presented an interesting aspect of this guild. Taxa affiliated to *Halobacteriaceae* are known mesophilic or moderate thermophilic halophile, able to utilize various organic substrates, can withstand slightly acidic to alkaline pH and reduce sulfur to sulfides (Robinson et al., 2005; Oren, 2014a; Thombre et al., 2016). Relative abundance of organic carbon in BS than in GR along with presence of methanogens capable of producing local alkalinity may be attributed to the presence of *Halobacteriaceae* in BS rocks. Co-occurrence network indicating the closeness of anaerobic hydrogenotrophic methanogens (*Methanobacterium* and *Methanocalculus*) with *Halobacteriaceae* members constituting the network hotspot possibly suggest a metabolic interrelation allowing co-occurrence of these two archaeal taxa. These hydrogenotrophic methanogens are known to have a lower threshold for hydrogen (geogenic and/or biogenic) concentration (compared to their acetoclastic counterparts) and harbor enzymes responsible for carbonate precipitation, thus allowing an increase in alkalinity of the local environment. Such a local rise in pH could possibly allow the alkaliphilic members to thrive (Smith and Ferry, 1999; Ussler and Paull, 2008). Absence of these methanogens along with lower levels of organic carbon in GR horizon might be one of the major factors limiting the presence of haloarchaeal members in deeper granitic horizons. Although most of these archaeal taxa have been reported previously in various deep biosphere investigations (Bomberg et al., 2015; Purkamo et al., 2015; Lau et al., 2016; Ijiri et al., 2018; Leandro et al., 2018; Hoshino and Inagaki, 2019), their abundance and association are important findings shading light on the potential roles of these extremophiles on deep biosphere biogeochemical carbon, sulfur cycles and energy flows (Purkamo et al., 2015; Jungbluth et al., 2016; Ijiri et al., 2018; Hoshino and Inagaki, 2019).

Archaeal microbiome within the organic carbon lean, basement Archaean granite rocks is distinct and constitute predominantly by acetoclastic methanogens (*Methanomicrobia*), ammonia oxidizing autotrophic archaea (SAGMCG-1, AK59, FHMa11 terrestrial group) and other taxa previously reported from diverse organic carbon deprived, deep crystalline environment with low oxygen (Takai et al., 2001; Kendall and Boone, 2006; Sørensen and Teske, 2006; Schneider et al., 2013; Oren, 2014b; Lauer et al., 2016; Wong et al., 2017; Purkamo et al., 2018; Smith et al., 2019). The presence of acetoclastic methanogenic *Methanomicrobia* as a dominant archaea in GR could be explained by relative higher concentration of acetate in these rocks, compared to BS (data not shown) as well as a lack of hydrogen generating minerals (e.g., olivine and pyroxene). The network consisting of these acetoclastic taxa (*Methanosaeta*, *Methanolinea*) shows their close proximity with anaerobic methane oxidizers (ANME-3) and major geological parameters, which had an increasing trend with depth. Though *Methanosaeta* and *Methanolinea* are both methanogenic in nature, the close phylogenetic relationship of *Methanosaeta* with anaerobic-methane oxidizers (ANME-1 and ANME-2) and the presence of the genes for the CO_2_ reduction pathway raises the possibility that *Methanosaeta* could have the capacity for the oxidation of methane (Smith and Ingram-Smith, 2007; Knittel and Boetius, 2009). It was initially thought that *Methanosaeta* was obligatory acetoclastic methanogen, but it was later reported that *Methanosaeta* can make direct electrical connections with *Geobacter* species, accepting electrons for the reduction of carbon dioxide to methane (Rotaru et al., 2014; Zhao et al., 2015). Archaeal genus ANME-3, which is also a part of this cluster, is not only capable of anaerobic oxidation of methane but also uses methane as one of the main carbon sources (Niemann et al., 2006). ANME-3 was earlier observed in close association with bacterial genus *Desulfobulbus* in the sulfide rich zones and there are evidences that confirm that *Desulfobulbus* can thrive on methane derived carbon (Niemann et al., 2006; Cui et al., 2015; Winkel et al., 2018). Interestingly, these three archaeal genera (*Methanosaeta*, *Methanolinea*, and ANME-3) also show negative correlation with TOC, which further supports the possibility of autotrophic lifestyle in the deeper rock cores (Knittel and Boetius, 2009). This sub-network consisting of both methanotrophs and methanogens not only indicates that methane might be an important driver of life in the deeper horizons of Deccan but also supports the possibilities of SLiME (Stevens and McKinley, 2000; Nealson et al., 2005) in the deep terrestrial subsurface of Deccan traps. Metagenome function is further predicted from 16S rRNA gene sequences using PICRUSt. PCA biplot constructed on the basis of the relative abundance of different predicted metabolisms corroborate the clustering discussed previously. Comparable trends obtained from different statistical analysis (on the basis of taxonomy and predicted community functions) determine that taxonomy of different archaeal groups in a particular metagenome reflects the archaeal community function.

In conclusion, this is the first detailed study on archaeal diversity and their niche-specific diversity shift in subterranean igneous provinces of the seismically active Koyna–Warna region of Deccan traps. Our results demonstrate a distinct partitioning of archaeal communities across the basaltic and granitic horizons corroborating the characteristic geochemical/physical conditions of these two horizons. Basaltic horizon harbors thermoacidophilic, chemolithotrophic, and organotrophic archaeal groups known to occupy environments with geothermal/volcanic/tectonic activities and with significant contributions in iron and sulfur cycles. A close association of halophilic taxa and hydrogenotrophic methanogenic archaea is noted. The deeper, more oligotrophic granitic horizons, in contrast, is dominated by chemoautotrophic archaea present in close association with acetoclastic methogenic taxa. The study provides a detailed report of archaeal communities from deep crystalline crustal environment highlighting the potential role of acidophilic, iron-oxidizing, sulfur-respiring, halophilic, methanogenic, methane/ammonia oxidizing archaea in carbon and energy flows in deep terrestrial crust.

## Author Contributions

AD carried out the microbiological analysis, statistical analysis, data organization, and manuscript preparation. SDG carried out the geochemical analysis and assisted in statistical analysis. AD and AG performed sub-coring of rocks, metagenomic DNA extraction, and bioinformatics pipeline optimization. AD, AG, and JS carried out qPCR analysis. HB assisted in data interpretation and manuscript preparation. SR organized sample collection and gave inputs about geophysical/geochemical characterization of Deccan lava flow and Koyna–Warna seismic zone. AM gave inputs about conducting different geochemical analysis. PS conceived the study, designed experiments, compiled and analyzed the data, wrote the manuscript, and done overall mentoring.

## Conflict of Interest Statement

The authors declare that the research was conducted in the absence of any commercial or financial relationships that could be construed as a potential conflict of interest.

## Footnotes

1 https://vamps.mbl.edu/resources/primers.php
