# Supplementary material for: Archaeal Communities in Deep Terrestrial Subsurface Underneath the Deccan Traps, India
Source: Front Microbiol. 2019 Jul 16;10:1362. doi: 10.3389/fmicb.2019.01362 (PMC6646420; doi:10.3389/fmicb.2019.01362)
Supplement: Supplementary file 1 [file Data_Sheet_1.PDF]

Table 1. List of samples recovered from different scientific boreholes **(A)** Samples recovered from borehole at Phansavale **(B)** Samples recovered from borehole at Ukhalu

(a) **Scientific Borehole location: Phansavale**

Latitude: N17° 09.017' **Longitude:** E073° 40.058' **Altitude=**131 m

Sample Name	Depth (meters below surface)	Nature
PV4	106.4	Basalt
PV2	322.41	Basalt
PV5	423.5	Basalt
PV7	512.7	Transition zone
PV8	521.8	Migmatitic gneiss
PV13	898.33	Granite

(b) **Scientific Borehole location: Ukhalu**

Latitude: N 17° 07.552' **Longitude:** E 073° 52.148' **Altitude=**567 m

Ukhalu	Depth (meters below surface)	Nature
U9	59.6	Massive Basalt
U8	262.15	Amygdaloidal Basalt
U4	937.9	Granite gneiss
U3	1050.7	Granite
U14	1402.69	Granite

Table 2. Quantitative PCR and amplicon sequencing primer details

Gene	Primer Name	Primer Sequence (5'-3')	Source
Archaeal 16S rRNA gene (qPCR)	A344f	ACGGGGCGCAGCAGGCGCGA	Bano *et al.* 2004
	Ar774r	CCCGGGTATCTAATCC	Barns *et al.* 1994
mcrA (qPCR)	ME1	GCMATGCARATHGGWATGTC	Hales *et al.* 1996
	ME3	TGTGTGAAWCCKACDCCACC	Nyyssönen *et al.* 2012
Archaeal 16S rRNA gene (Amplicon sequencing)	517F	GCCTAAAGCATCCGTAGC<br>GCCTAAARCGTYCGTAGC<br>GTCTAAAGGGTCYGTAGC<br>GCTTAAAGNGTYCGTAGC<br>GTCTAAARCGYYCGTAGC	VAMP website (https://vamps.mbl.edu/resources/primers.php)
	958R	CCGGCGTTGANTCCAATT	

Table 3. Geochemistry of different rock samples

		U9	U8	PV4	PV2	PV5	U4	PV7	PV8	U3	PV13	U14
		BS	BS	BS	BS	BS	GR	TZ	GR	GR	GR	GR
Normalized Depth (m)		507.4	304.9	24.6	-191.4	-292.1	-370.9	-381.0	-390.8	-483.7	-767.33	-835.7
Actual Depth (m)		59.6	262.2	106.4	322.4	423.5	937.9	512.0	521.8	1050.7	898.33	1402.69
Oxides (%)	SiO_2	34.4	34.9	35.6	40.4	26.5	45.6	67.9	59.8	53.3	82.573	61.0
	Al_2O_3	9.6	9.7	6.9	9.3	6.5	11.4	11.1	12.3	6.7	6.02	15.6
	K_2O	0.1	0.3	0.4	1.0	1.5	3.3	8.8	1.9	9.2	10.228	8.5
	CaO	16.0	12.3	10.3	10.8	12.5	7.0	3.0	3.7	8.7	0	1.4
	TiO_2	3.9	3.6	4.9	3.4	4.8	2.6	0.5	1.1	1.6	0.007	1.1
	V_2O_5	0.1	0.1	0.1	0.1	0.2	0.0	0.0	0.0	0.0	0	0.0
	Fe_2O_3	31.7	34.0	35.9	29.7	41.1	24.8	6.6	12.7	17.9	0.254	8.6
Carbon (mg/kg)	TOC	48.1	46.0	34.2	31.0	25.5	41.2	16.9	9.3	35.6	4.63	33.0
	TIC	98.3	93.2	101.2	95.1	87.8	82.6	92.6	49.1	75.7	33.56	70.9
	TC	149.3	143.0	137.2	124.0	113.0	127.7	111.9	61.7	116.0	40.02	106.1
Anions (mg/kg)	Cl^-	230.7	109.0	73.8	75.1	74	14.63	3.89	107.4	2.71	12.96	17.2
	NO_2^-	18.4	0.3	11.4	0.6	18.76	26.495	41.465	14.2	22.97	43.04	32.6
	SO_4^{2-}	0.0	834.5	0.0	0.0	230.62	12.51	168.72	978.7	17.04	281.01	17.94
	NO_3^-	1.4	67.0	22.8	0.0	4.05	13.12	40.95	53.1	10.95	42.96	51.06
	PO_4^{3-}	0.0	0.0	0.0	0.0	n.a.	n.a.	n.a.	19.2	n.a.	n.a.	n.a.
Elements (mg/kg)	Cd	1.4	0.8	1.8	2.3	3.1	5.17	2.5	10.0	18.03	5.4	5.98
	Ca	3046.6	4044.0	6427.5	6481.4	8756.2	4411.27	8683.35	8282.9	8302.07	2594.31	3485.88
	Cr	121.3	120.1	165.7	180.9	244.4	467.2	223.9	480.8	669.84	393.4	445.94
	Fe	1875.8	1845.7	2866.3	2925.7	3952.6	4338.44	3872.4	3945.7	5229.42	4698.6	4054.03
	K	3886.5	3945.7	7711.2	7816.5	10559.9	1234.43	10417.6	8220.1	1264.54	12192.5	1104.52
	Mg	6195.7	4575.7	4546.0	4890.8	6607.4	9120.07	6141.49	7800.8	12139.7	9557.3	7279.14
	Na	4995.7	4860.9	4410.4	3976.3	5371.9	6283.83	5958.28	3060.9	6121.05	5136.03	6526.19
Others	Alkalinity	58.5	19.5	19.5	19.5	94.25	151.63	12.95	948.8	256.96	962.47	547.23
	pH	6.8	6.8	6.7	6.8	7.0	7.2	7.3	7.9	7.9	8.0	8.0
	Ca:Na	0.6	0.8	1.5	1.6	1.6	0.7	1.5	2.7	1.4	0.5	0.5

N.B.: Unit of alkalinity is in mg/kg

Table 4. SIMPER analysis result displaying archaeal classes responsible for dissimilarity among basaltic and granitic zone.

Taxon	Av. dissim	Contrib. %	Cumulative %	Mean BS	Mean GR
Thermoplasmataceae	17.06	20.93	20.93	34.8	4.1
Ferroplasmaceae	9.704	11.91	32.84	21.3	2.72
Methanosaetaceae	6.891	8.454	41.29	3.14	14.2
SAGMCG-1_uncultured archaeon	6.595	8.091	49.38	3.41	15.7
AK59_Other	6.545	8.03	57.41	0.00689	13.1
FHMa11 terrestrial group_uncultured archaeon	5.621	6.896	64.31	0.647	11.4
Bathyarchaeota_uncultured archaeon	3.74	4.589	68.9	2.6	8.67
FHMa11 terrestrial group_Other	3.494	4.286	73.18	4.69	4.49
SAGMCG-1_Other	3.36	4.122	77.31	2.68	6.6
Methanobacteriaceae	3.01	3.692	81	6.22	0.368
Methanomicrobiaceae	2.521	3.093	84.09	4.92	0.526
Marine Benthic Group D and DHVEG-1	2.009	2.465	86.56	1.67	4.35
Sulfolobaceae	1.564	1.919	88.47	2.74	0.725
AMOS1A-4113-D04	1.198	1.47	89.94	2.4	0
Unassigned	1.179	1.447	91.39	1.95	4.02
Halobacteriaceae	1.006	1.234	92.62	2.01	0

Table 5. NSTI values of predicted metagenomes

Sample	Metric	Value
U9	Weighted NSTI	0.089635989
U8	Weighted NSTI	0.080967682
PV4	Weighted NSTI	0.290532267
PV2	Weighted NSTI	0.049122021
PV5	Weighted NSTI	0.148792134
U4	Weighted NSTI	0.32174087
PV7	Weighted NSTI	0.134274599
PV8	Weighted NSTI	0.178724258
U3	Weighted NSTI	0.282682553
PV13	Weighted NSTI	0.240479256
U14	Weighted NSTI	0.317209038

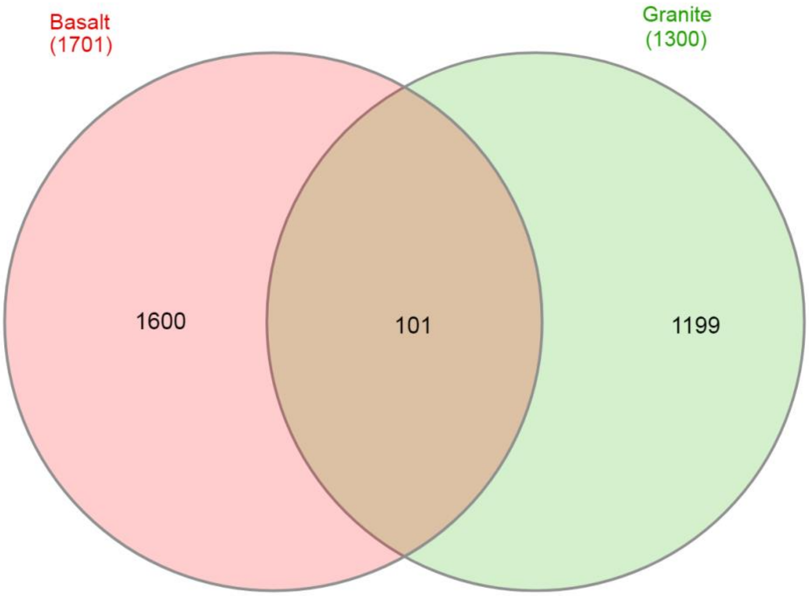

Figure 1. Overlap of archaeal OTUs across basalt and granitic horizons

References

Bano N, Ruffin S, Ransom B *et al*. Phylogenetic composition of Arctic Ocean archaeal assemblages and comparison with Antarctic assemblages. *Appl Environ Microbiol* 2004;**70**:781–9.

Barns SM, Fundyga RE, Jeffries MW *et al*. Remarkable archaeal diversity detected in a Yellowstone National Park hot spring environment. *Proc Natl Acad Sci* 1994;**91**:1609–13.

Hales BA, Edwards C, Ritchie DA *et al*. Isolation and identification of methanogen-specific DNA from blanket bog peat by PCR amplification and sequence analysis. *Appl Environ Microbiol* 1996;**62**:668–75.

Nyyssönen M, Bomberg M, Kapanen A *et al*. Methanogenic and sulphate-reducing microbial communities in deep groundwater of crystalline rock fractures in Olkiluoto, Finland. *Geomicrobiol J* 2012;**29**:863–78.
